# Supplementary figures and images for: Visual pursuit behavior in mice maintains the pursued prey on the retinal region with least optic flow
Source: eLife. 2021 Oct 26;10:e70838. doi: 10.7554/eLife.70838 (PMC8547958; doi:10.7554/eLife.70838)

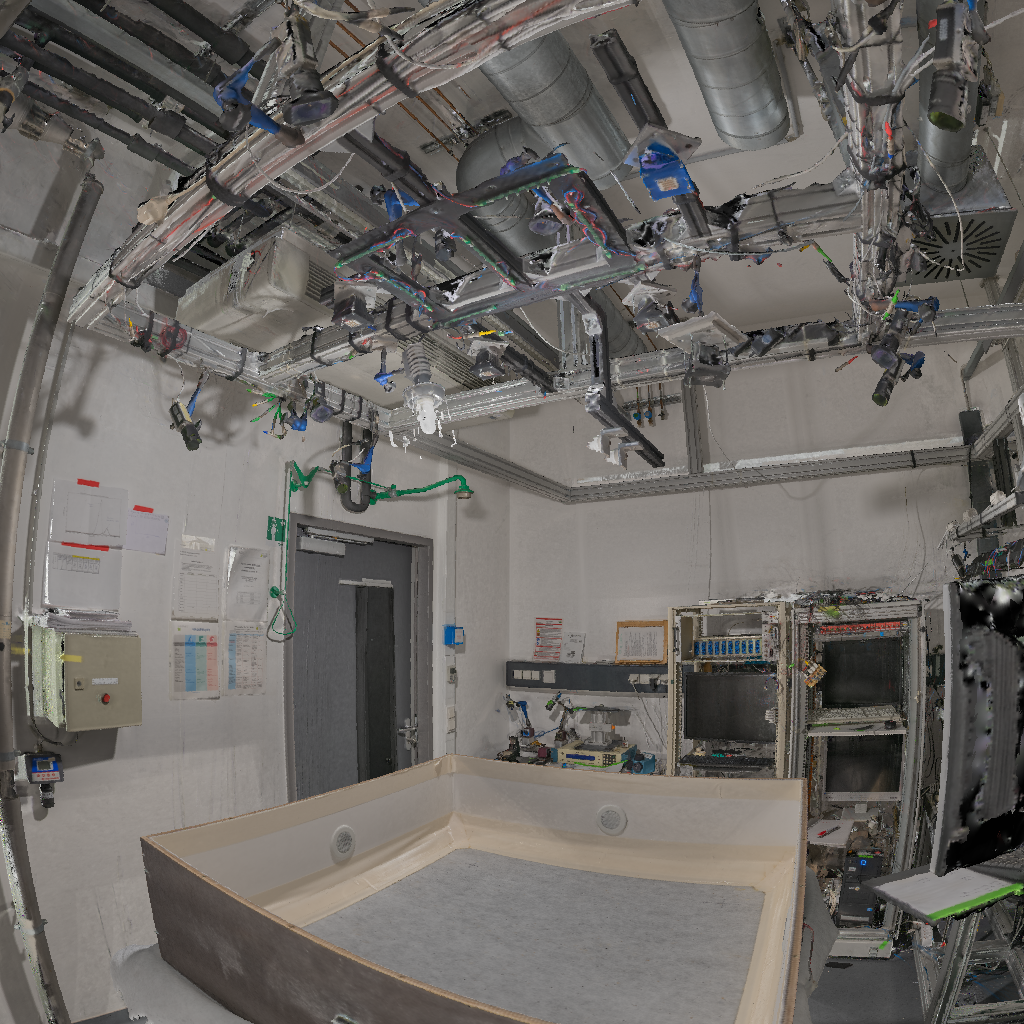

Supplement: Figure 1—source data 1. [file elife-70838-fig1-data1.zip › D/332.png]

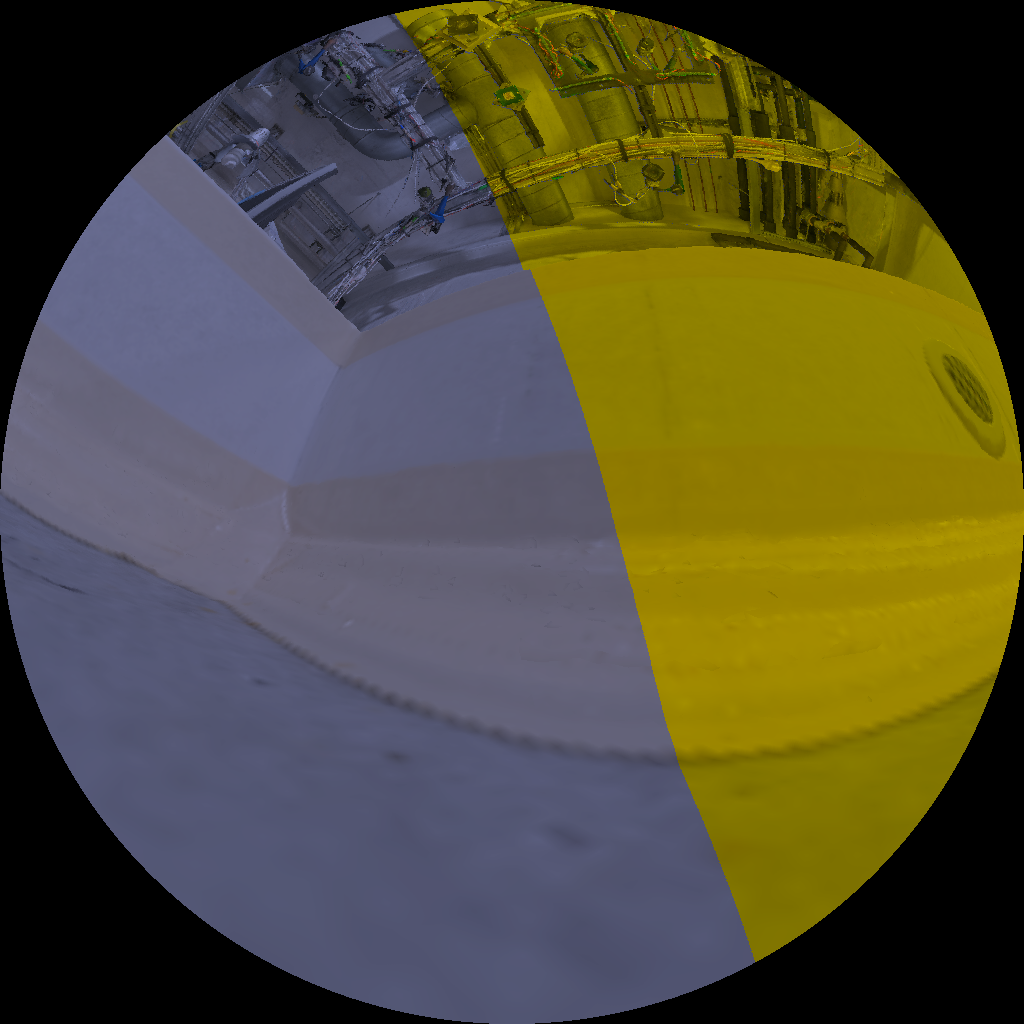

Supplement: Figure 1—source data 2. [file elife-70838-fig1-data2.zip › G/98354-left.png]

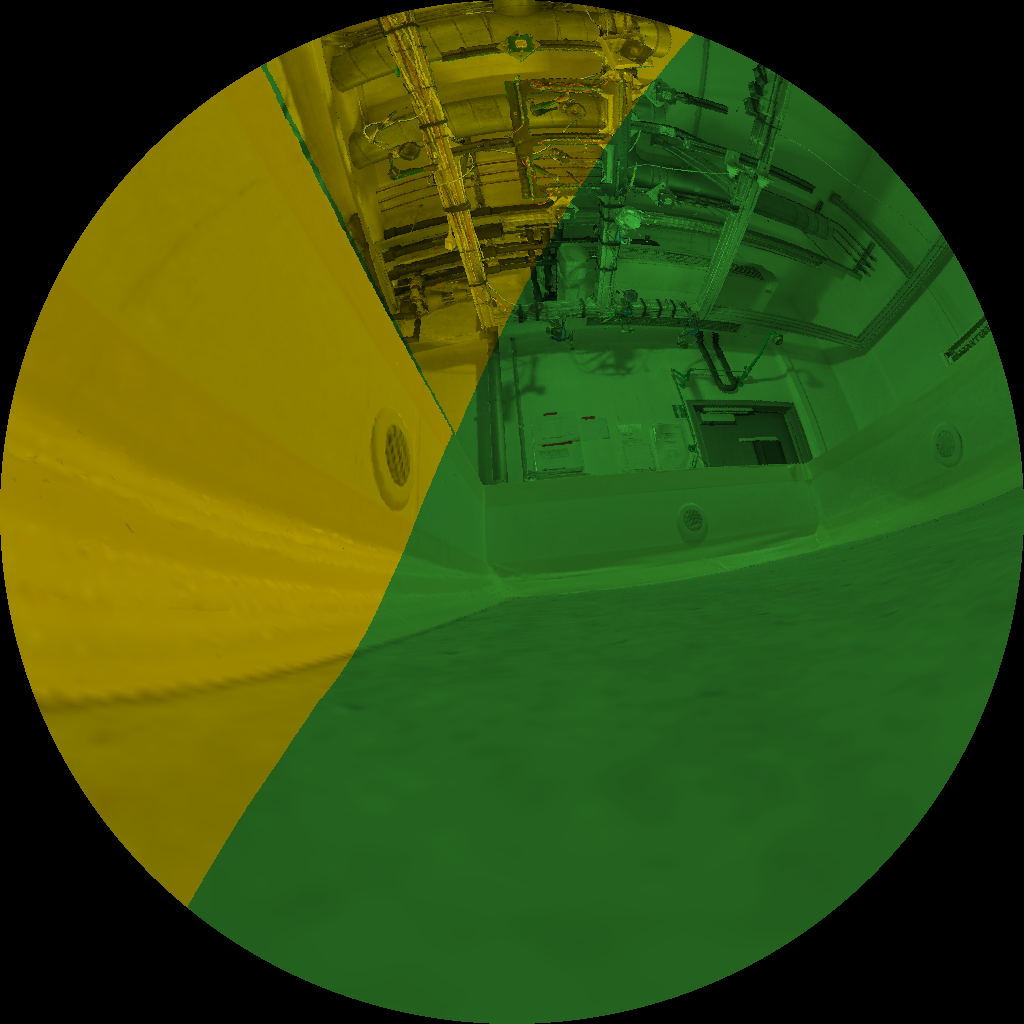

Supplement: Figure 1—source data 2. [file elife-70838-fig1-data2.zip › G/98354-right.png]

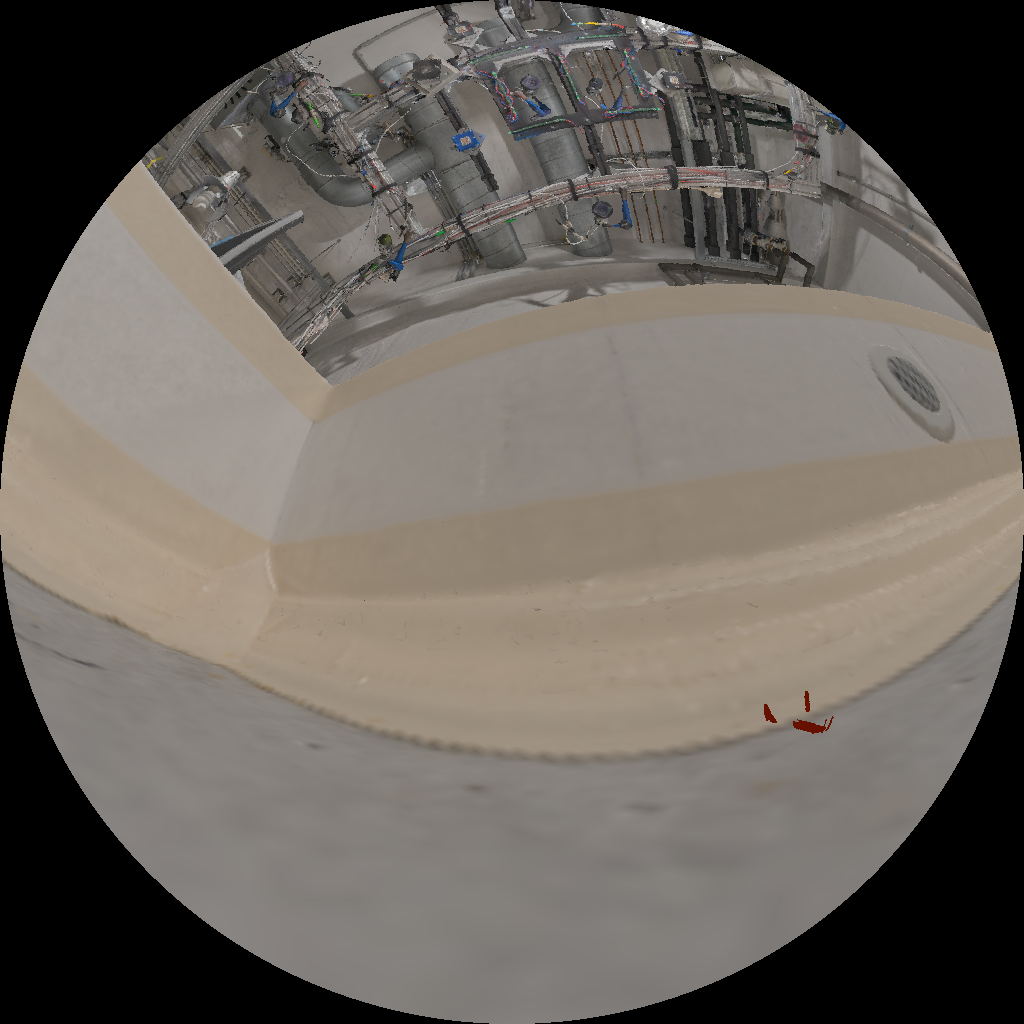

Supplement: Figure 1—source data 3. [file elife-70838-fig1-data3.zip › H/98349-left.png]

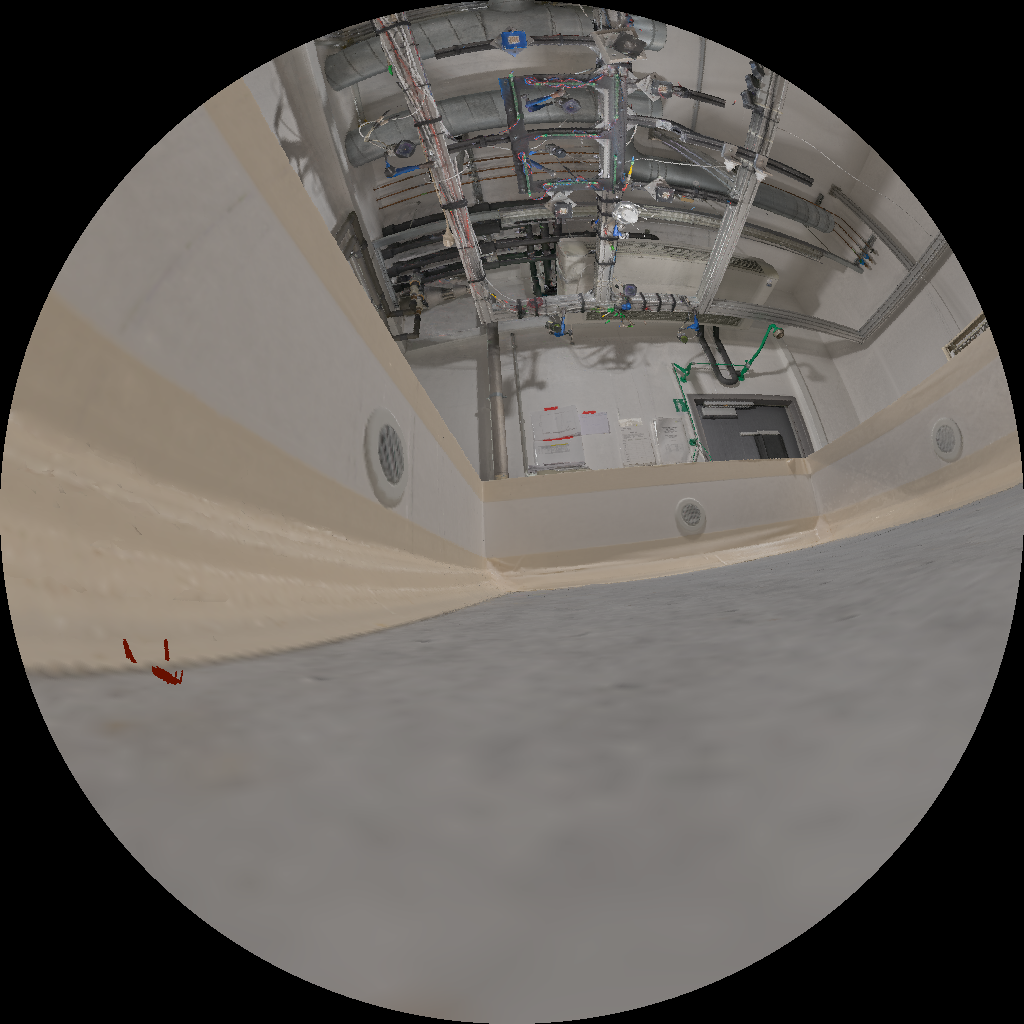

Supplement: Figure 1—source data 3. [file elife-70838-fig1-data3.zip › H/98349-right.png]

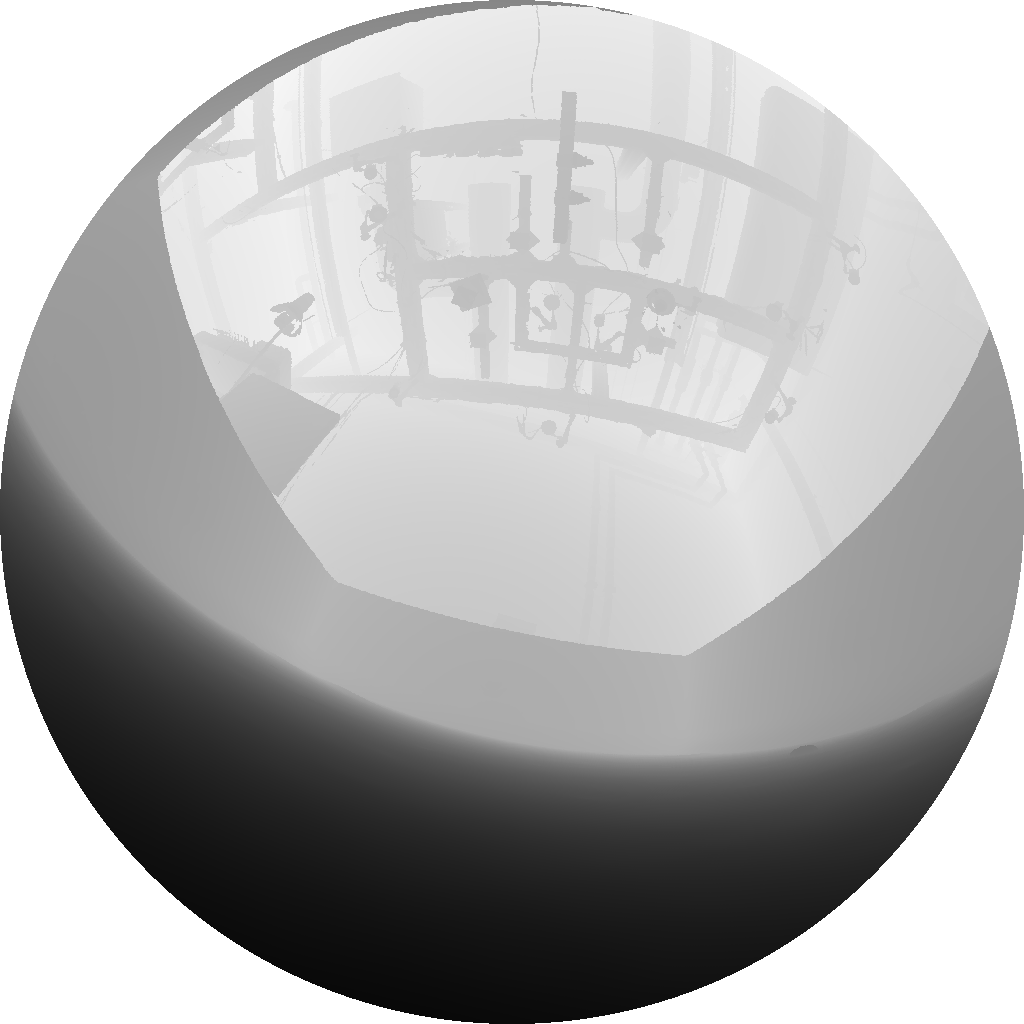

Supplement: Figure 1—source data 4. [file elife-70838-fig1-data4.zip › I/115444-left.png]

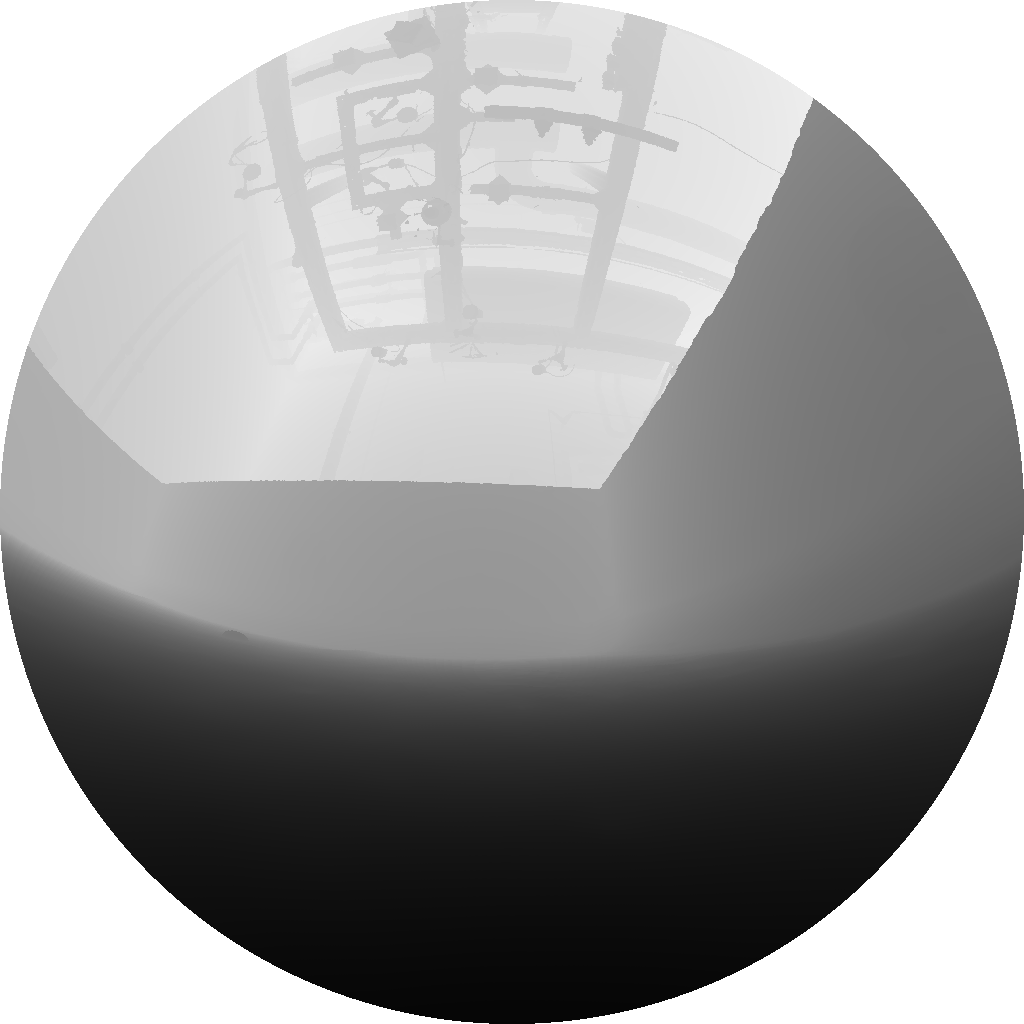

Supplement: Figure 1—source data 4. [file elife-70838-fig1-data4.zip › I/115444-right.png]

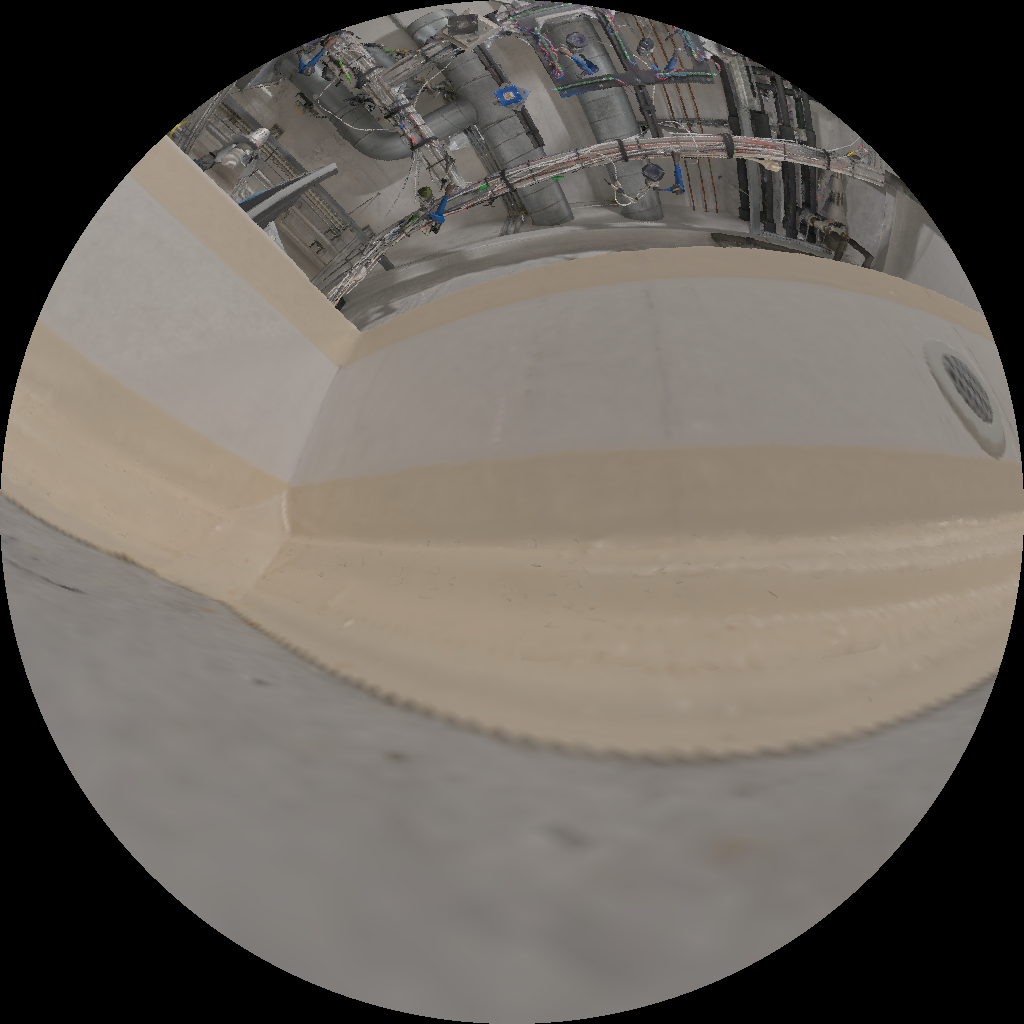

Supplement: Figure 1—source data 5. [file elife-70838-fig1-data5.zip › J/98349-left.png]

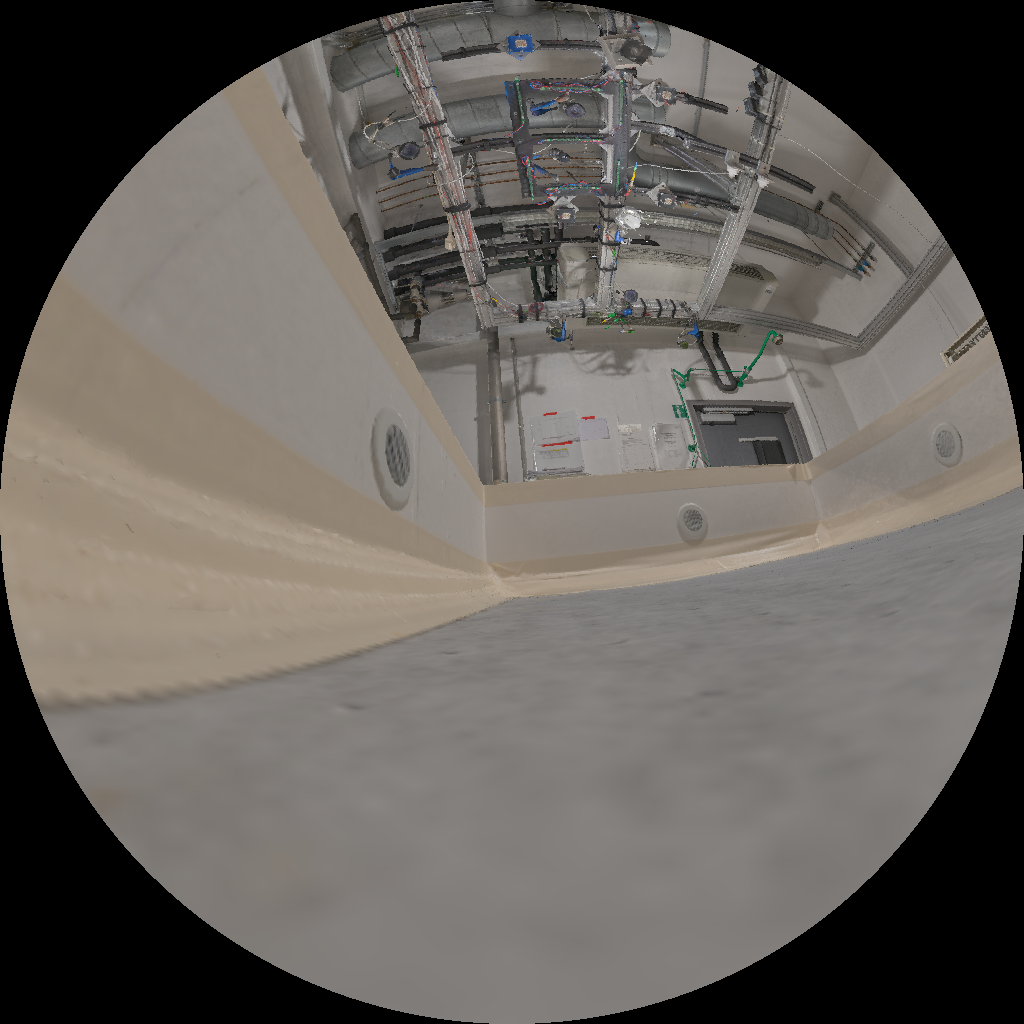

Supplement: Figure 1—source data 5. [file elife-70838-fig1-data5.zip › J/98349-right.png]

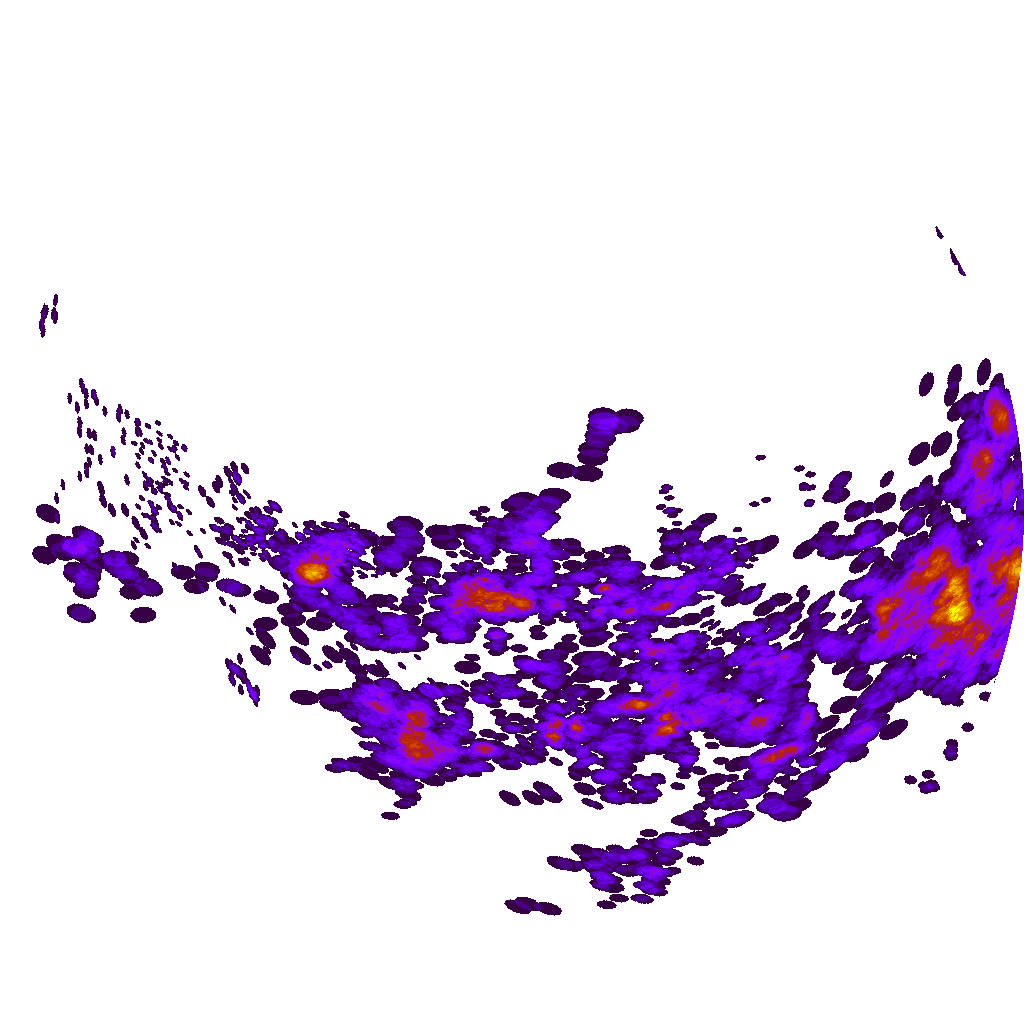

Supplement: Figure 2—source data 3. [file elife-70838-fig2-data3.zip › F/detect_allgr20-left.png]

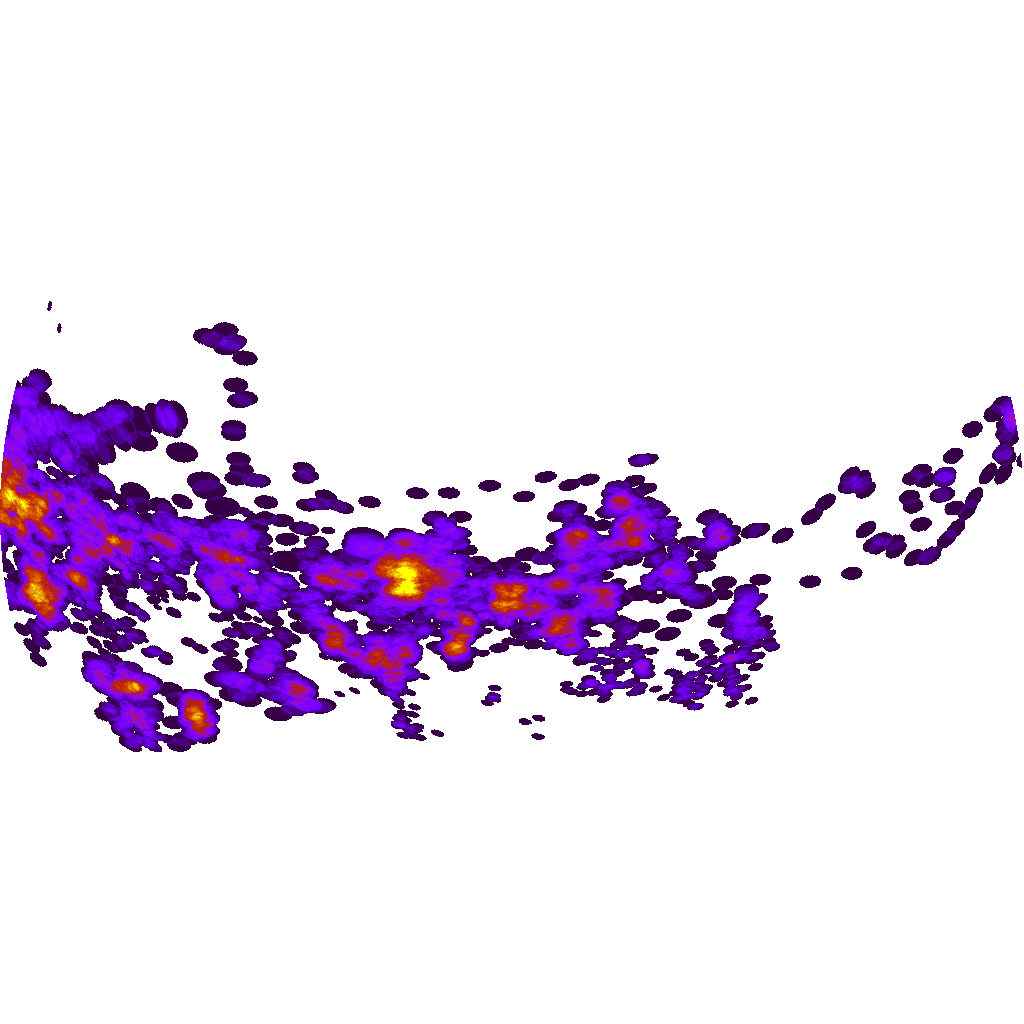

Supplement: Figure 2—source data 3. [file elife-70838-fig2-data3.zip › F/detect_allgr20-right.png]

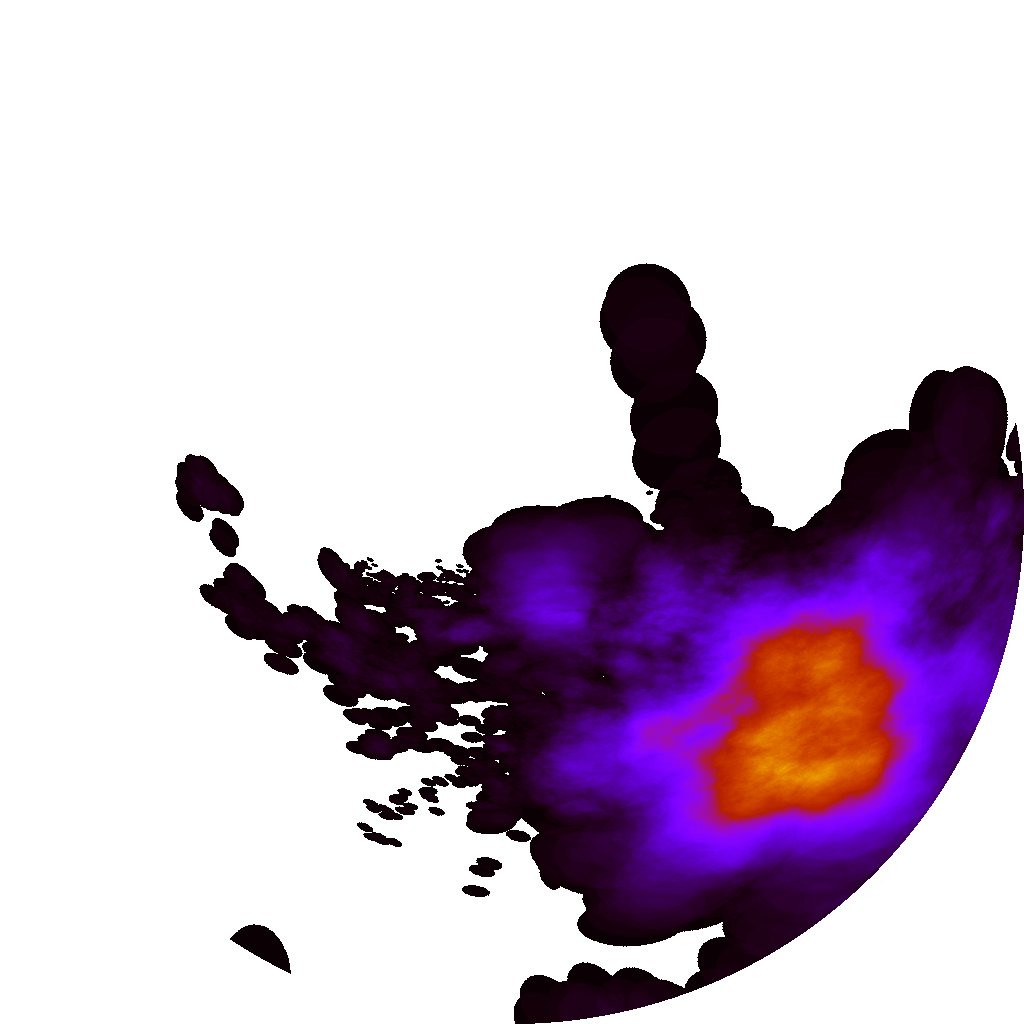

Supplement: Figure 2—source data 4. [file elife-70838-fig2-data4.zip › G/chase_allgr20-left.png]

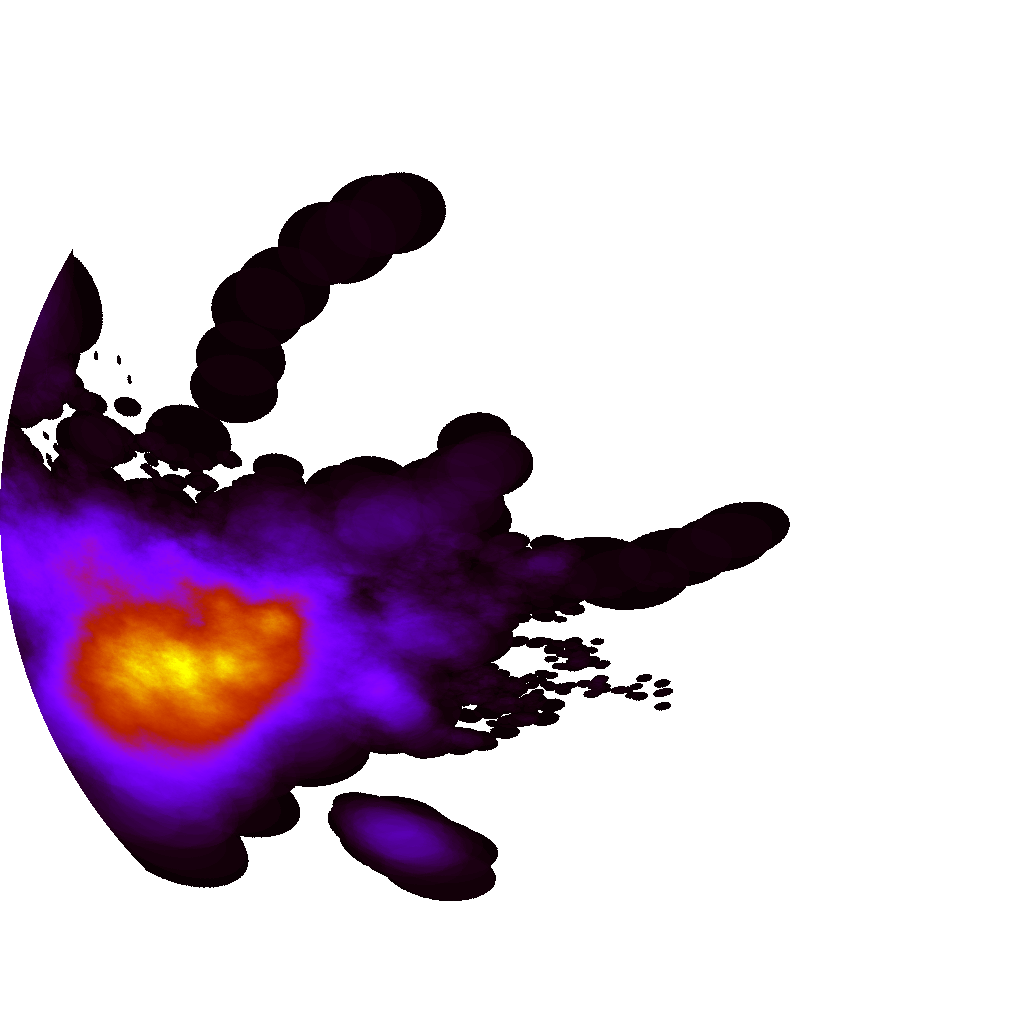

Supplement: Figure 2—source data 4. [file elife-70838-fig2-data4.zip › G/chase_allgr20-right.png]

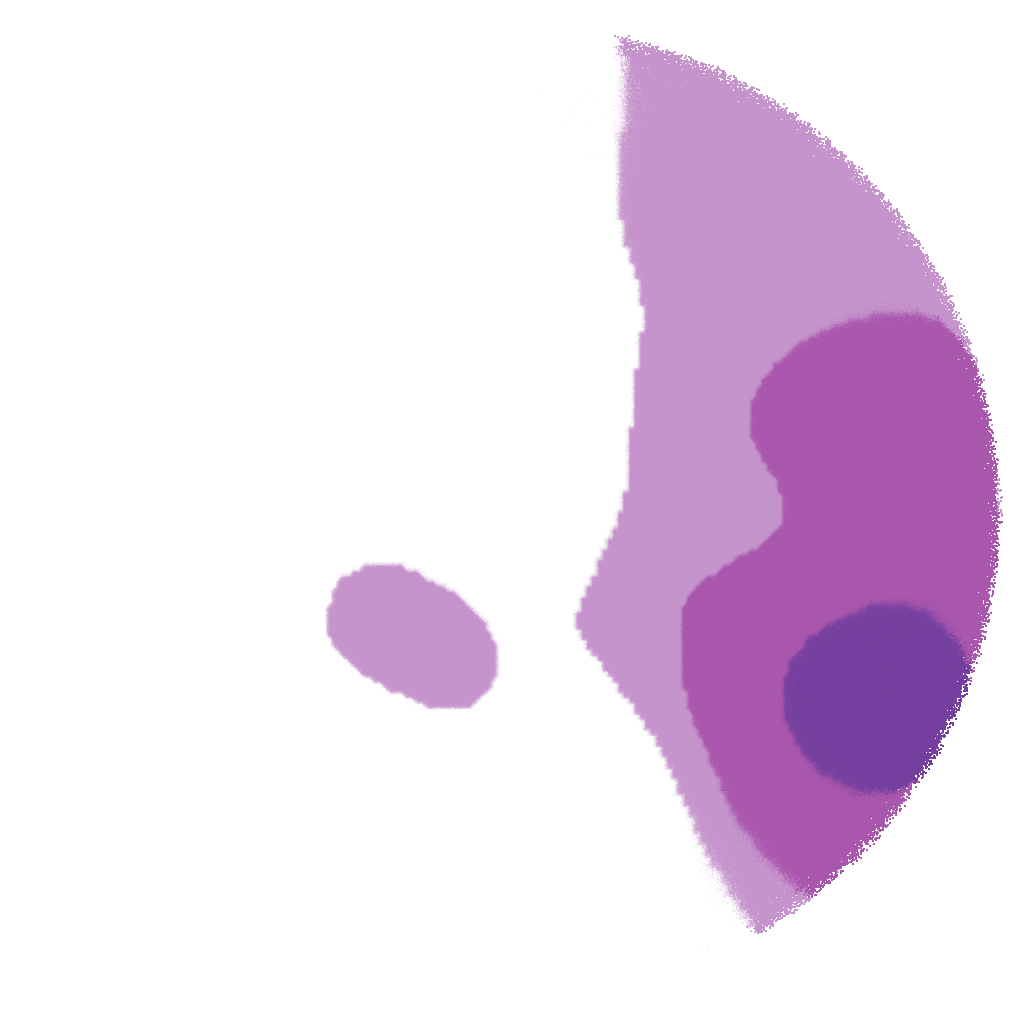

Supplement: Figure 3—source data 2. [file elife-70838-fig3-data2.zip › B/cornea_alpha_ganglion_cell_texture-colored-left.png]

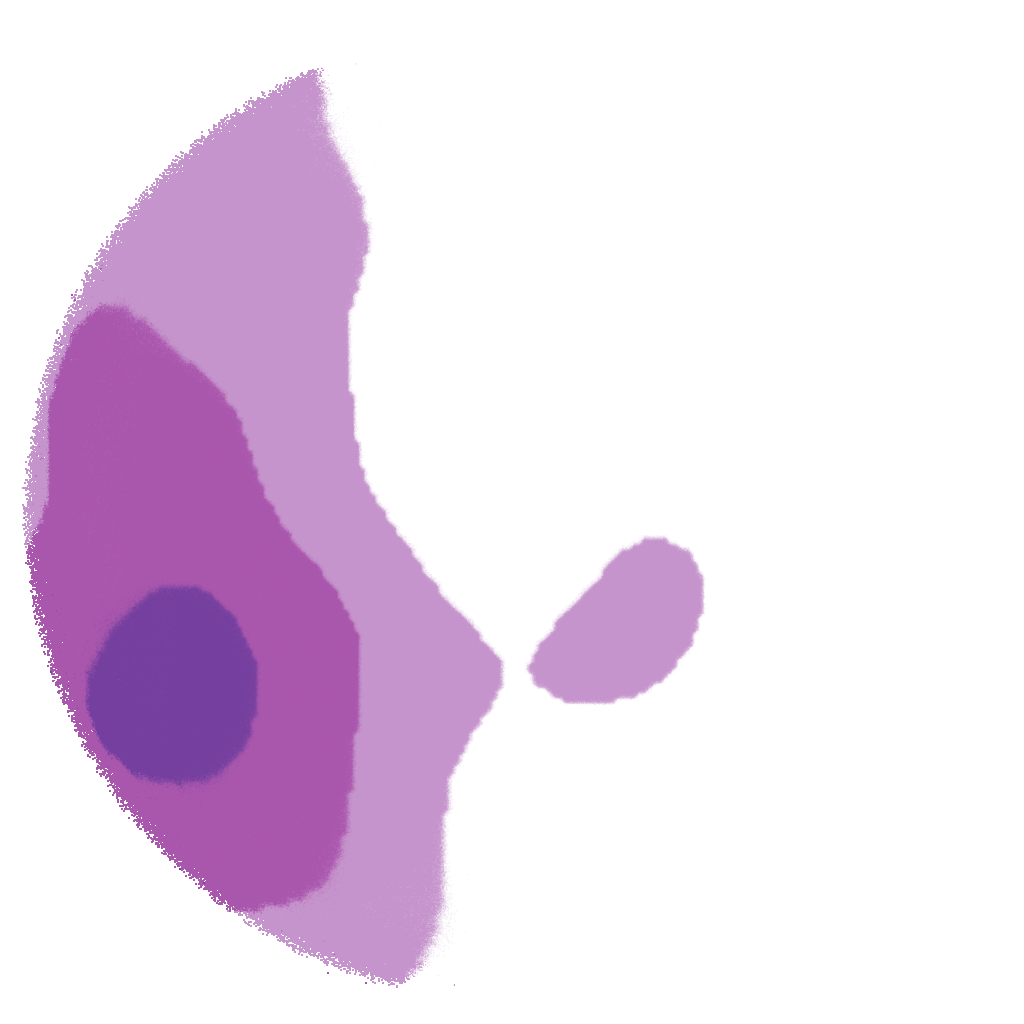

Supplement: Figure 3—source data 2. [file elife-70838-fig3-data2.zip › B/cornea_alpha_ganglion_cell_texture-colored-right.png]

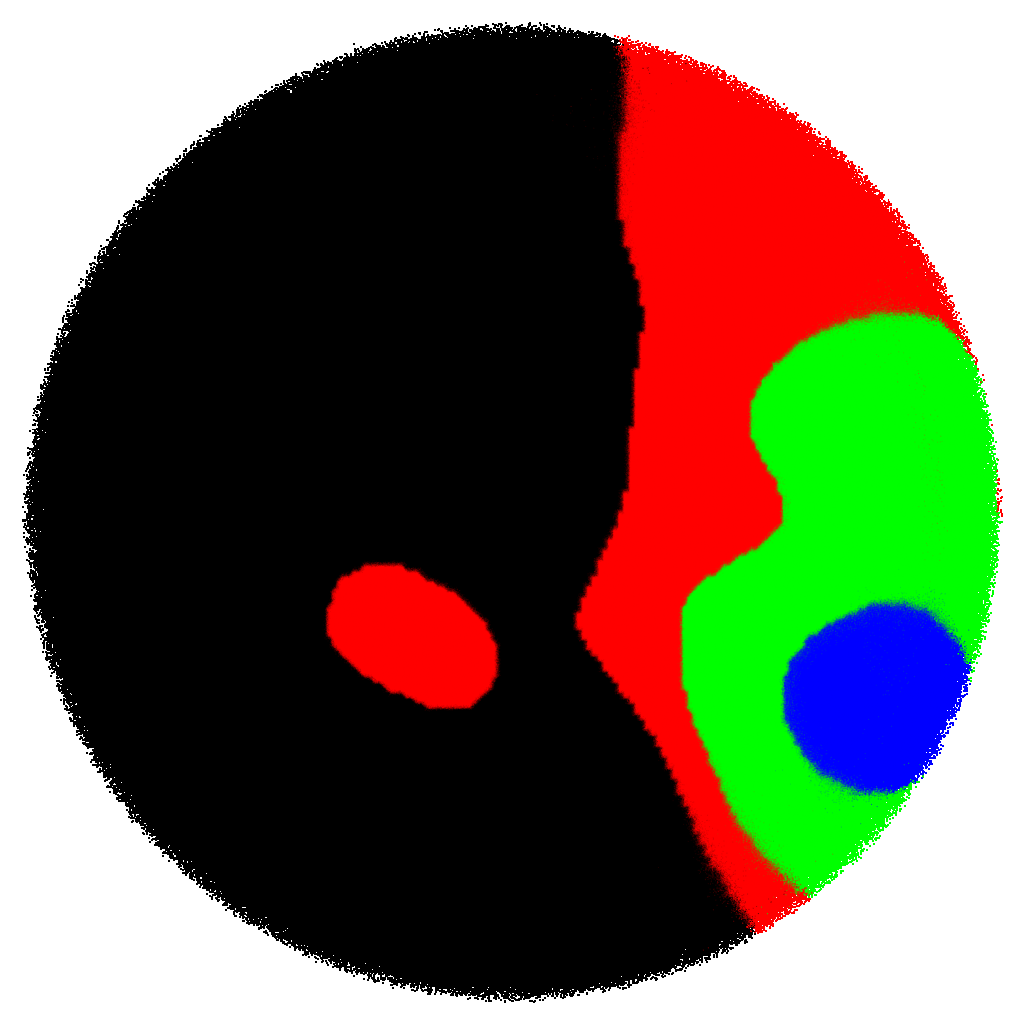

Supplement: Figure 3—source data 2. [file elife-70838-fig3-data2.zip › B/cornea_alpha_ganglion_cell_texture-left.png]

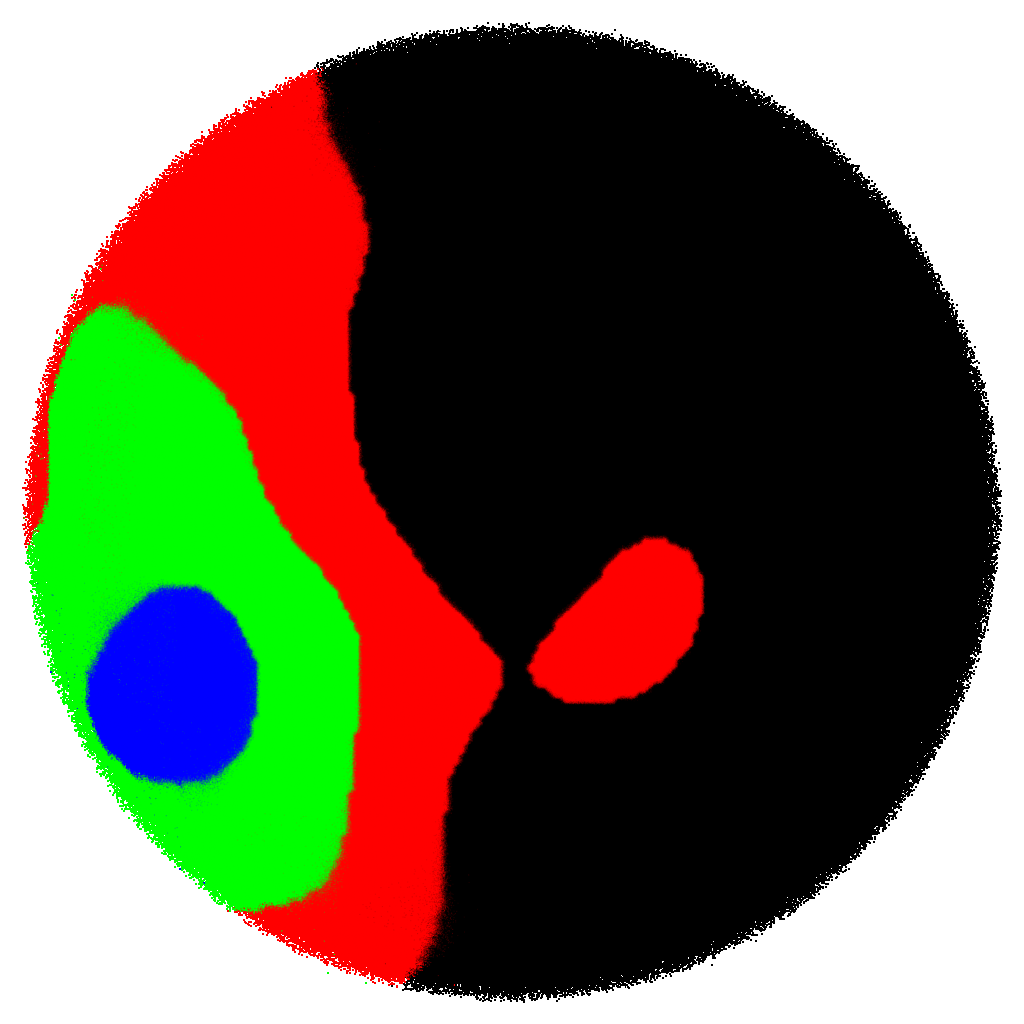

Supplement: Figure 3—source data 2. [file elife-70838-fig3-data2.zip › B/cornea_alpha_ganglion_cell_texture-right.png]

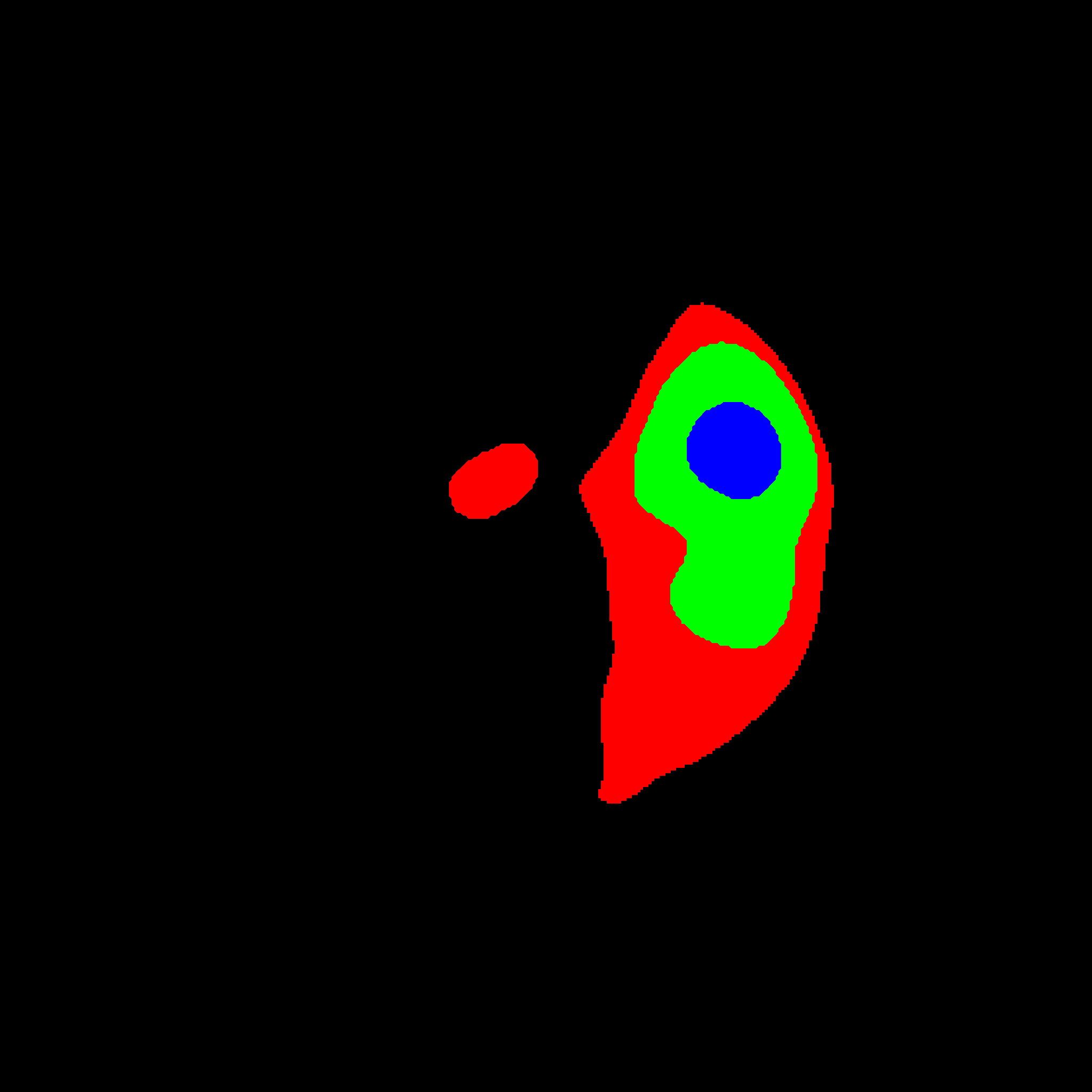

Supplement: Figure 3—source data 2. [file elife-70838-fig3-data2.zip › B/retinal_alpha_ganglion_cell_texture-left.png]

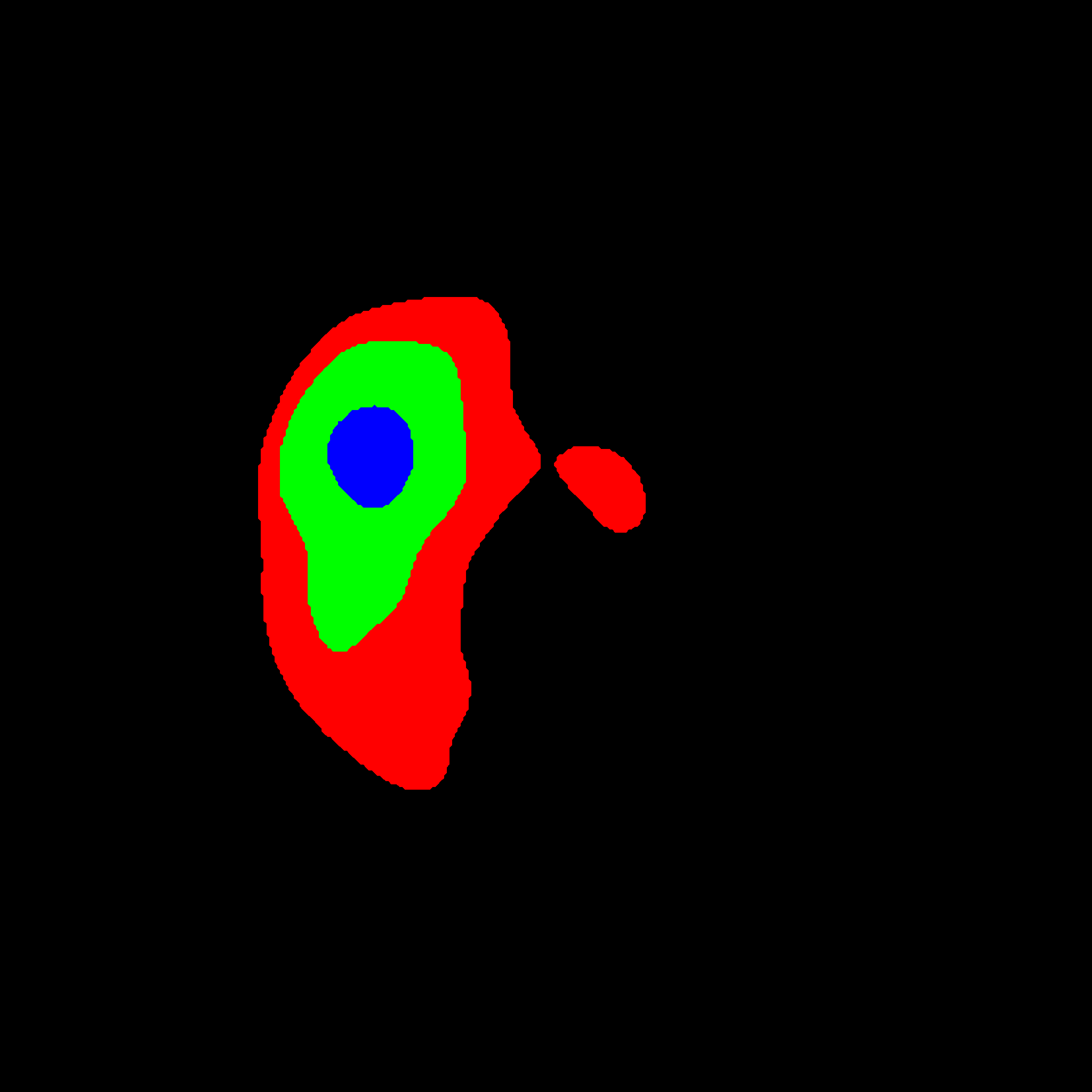

Supplement: Figure 3—source data 2. [file elife-70838-fig3-data2.zip › B/retinal_alpha_ganglion_cell_texture-right.png]

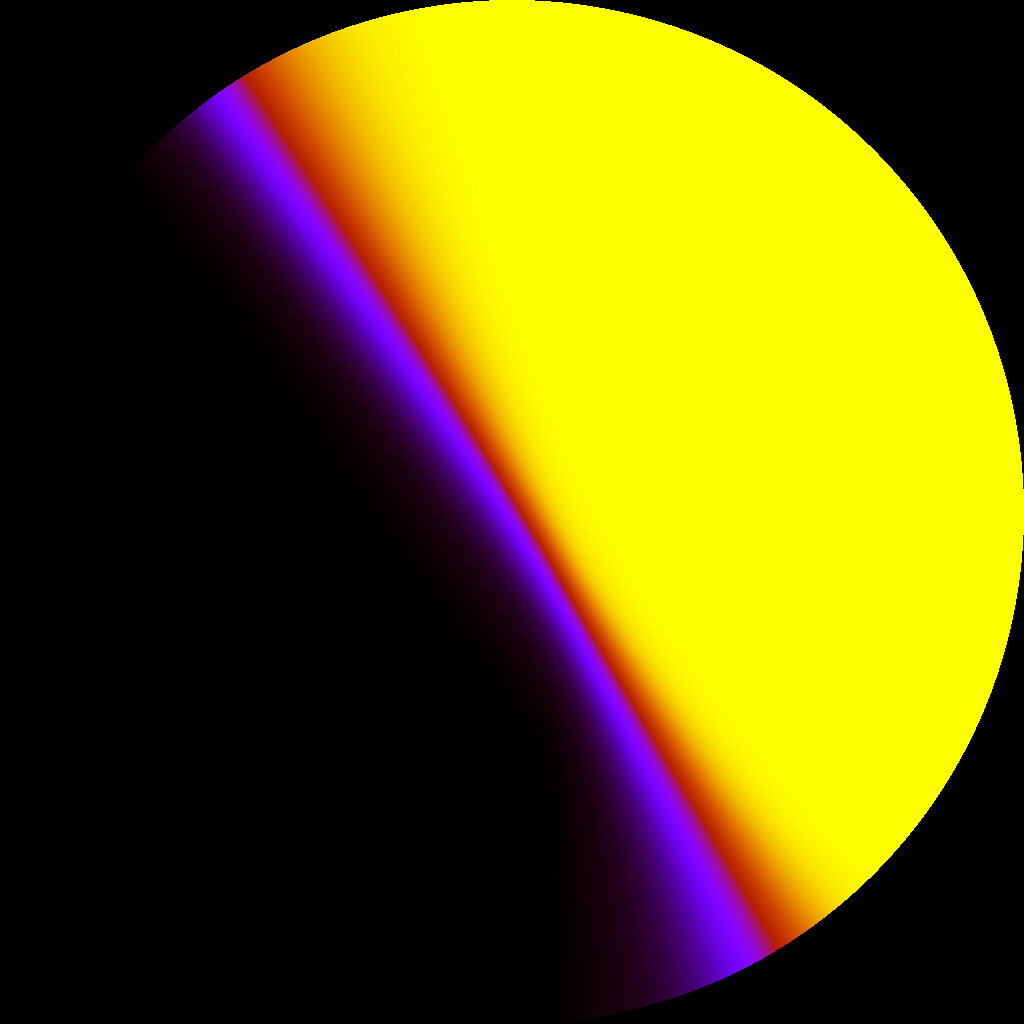

Supplement: Figure 4—source data 2. [file elife-70838-fig4-data2.zip › 4D/LeftEye.png]

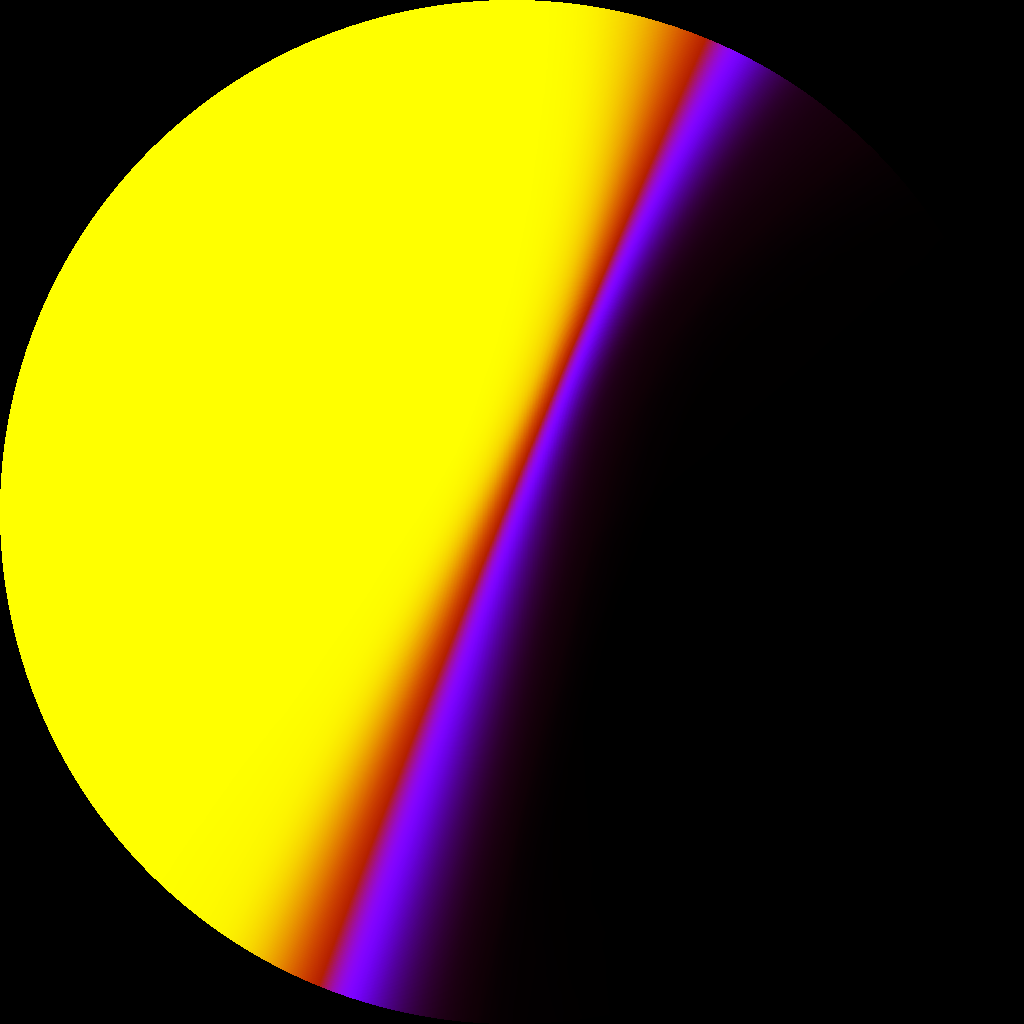

Supplement: Figure 4—source data 2. [file elife-70838-fig4-data2.zip › 4D/RightEye.png]

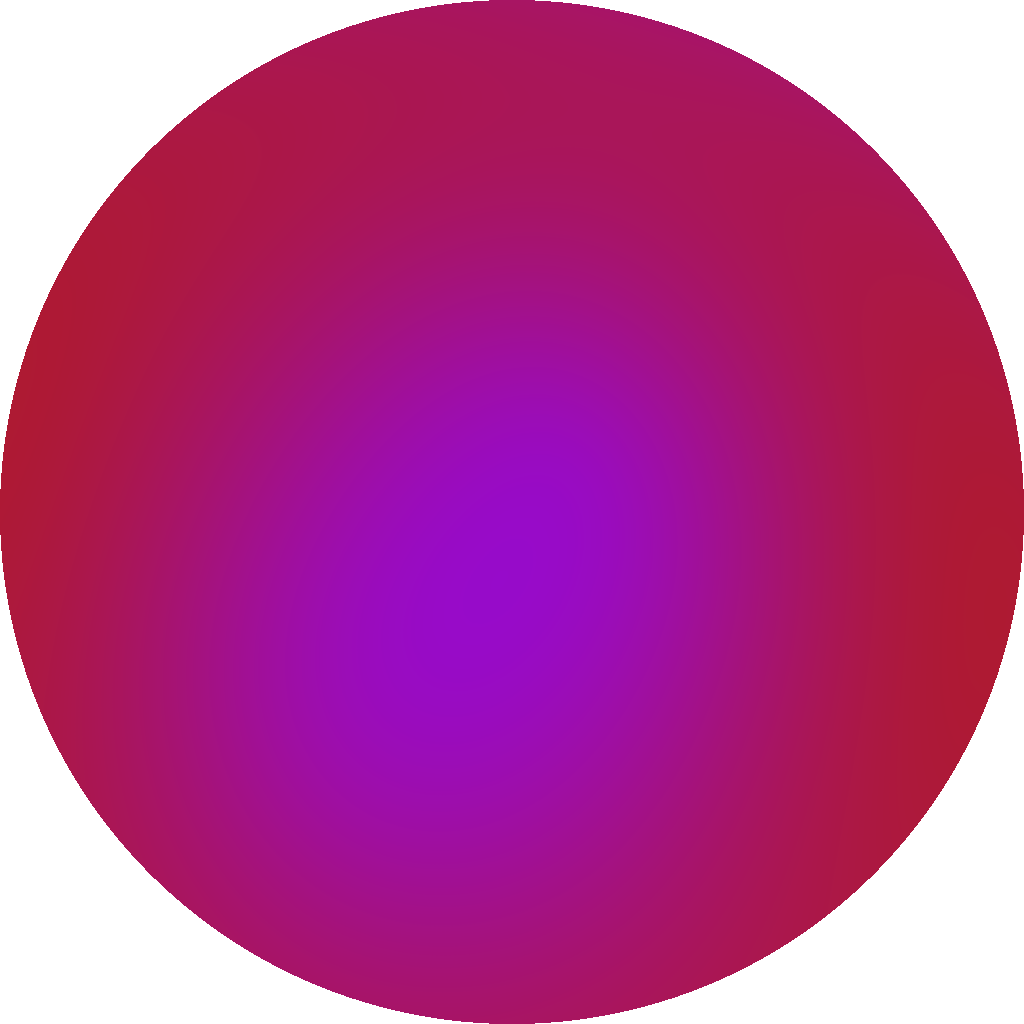

Supplement: Figure 4—source data 5. [file elife-70838-fig4-data5.zip › 4I/LeftEye.png]

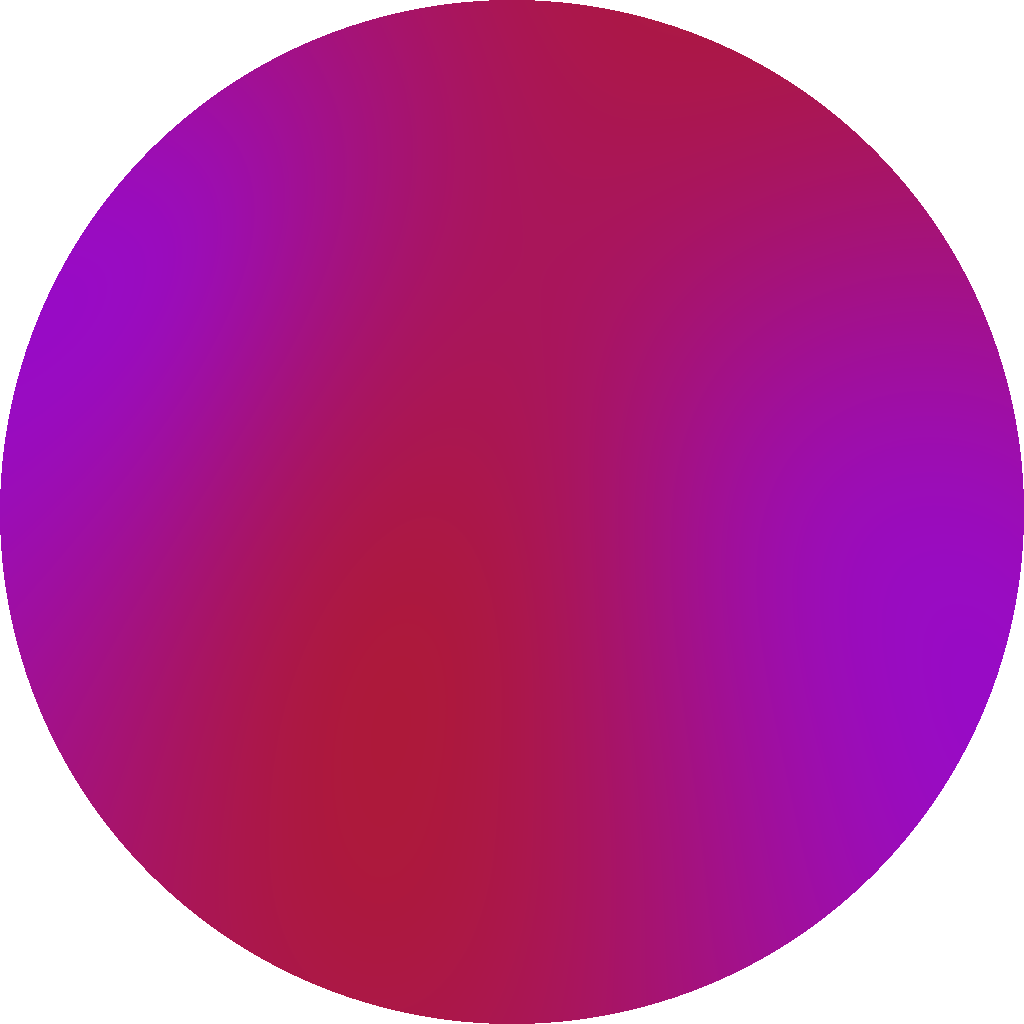

Supplement: Figure 4—source data 5. [file elife-70838-fig4-data5.zip › 4I/RightEye.png]

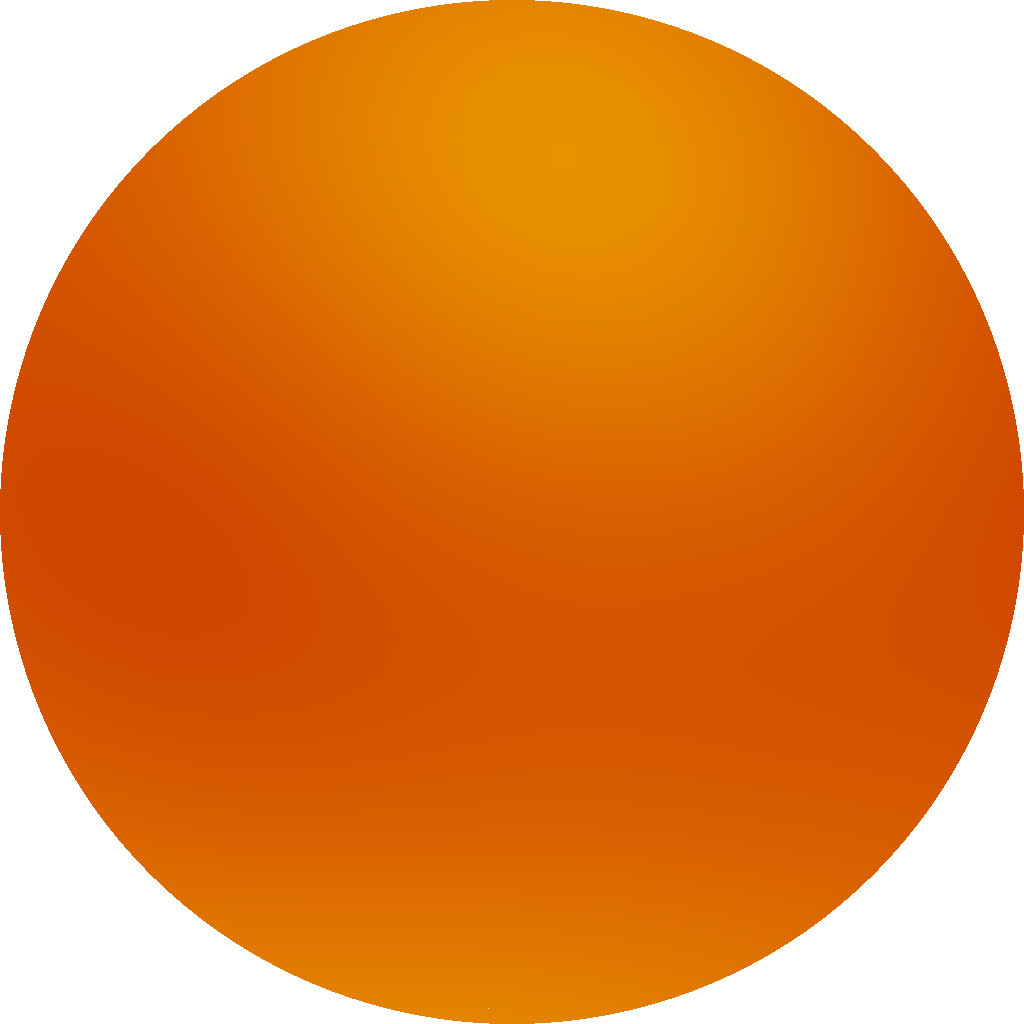

Supplement: Figure 4—source data 6. [file elife-70838-fig4-data6.zip › 4J/RightEye_LeftFixed.png]

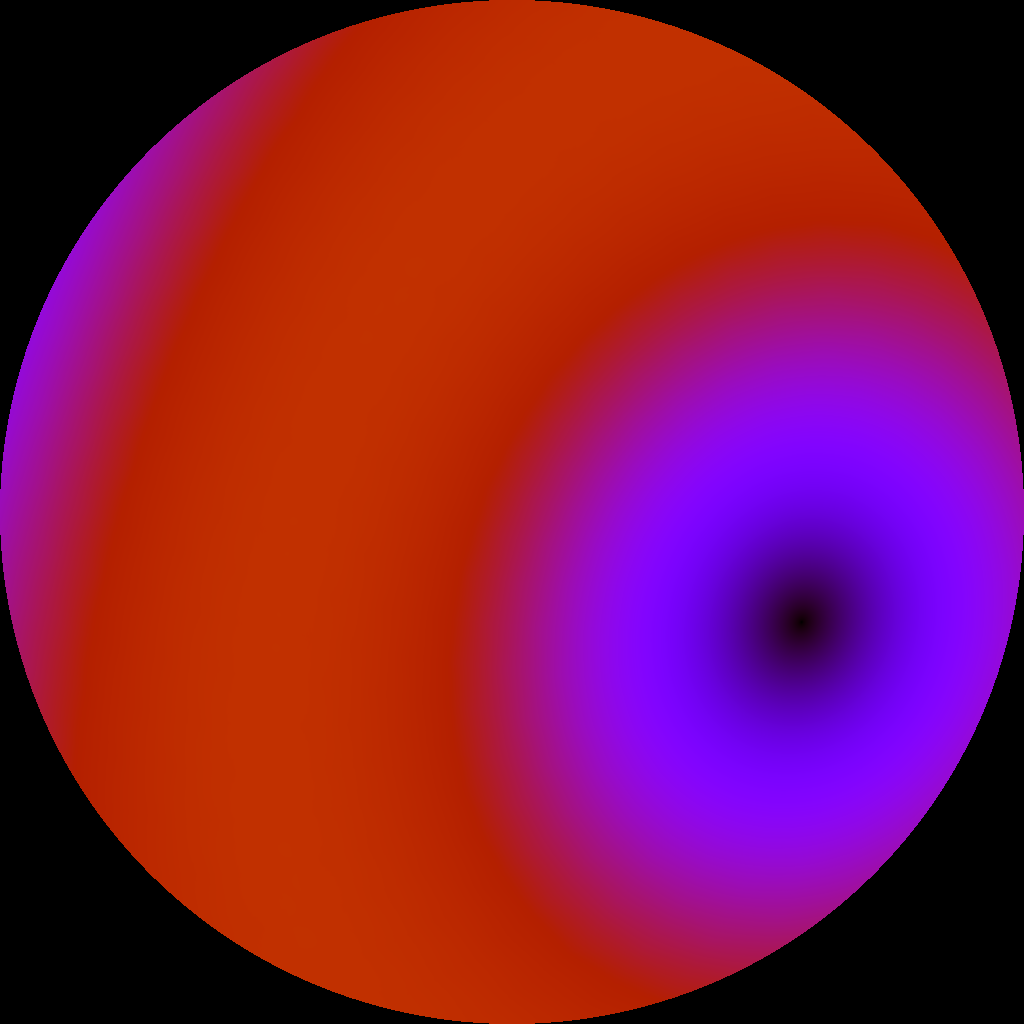

Supplement: Figure 6—source data 1. [file elife-70838-fig6-data1.zip › 6C/LeftEye.png]

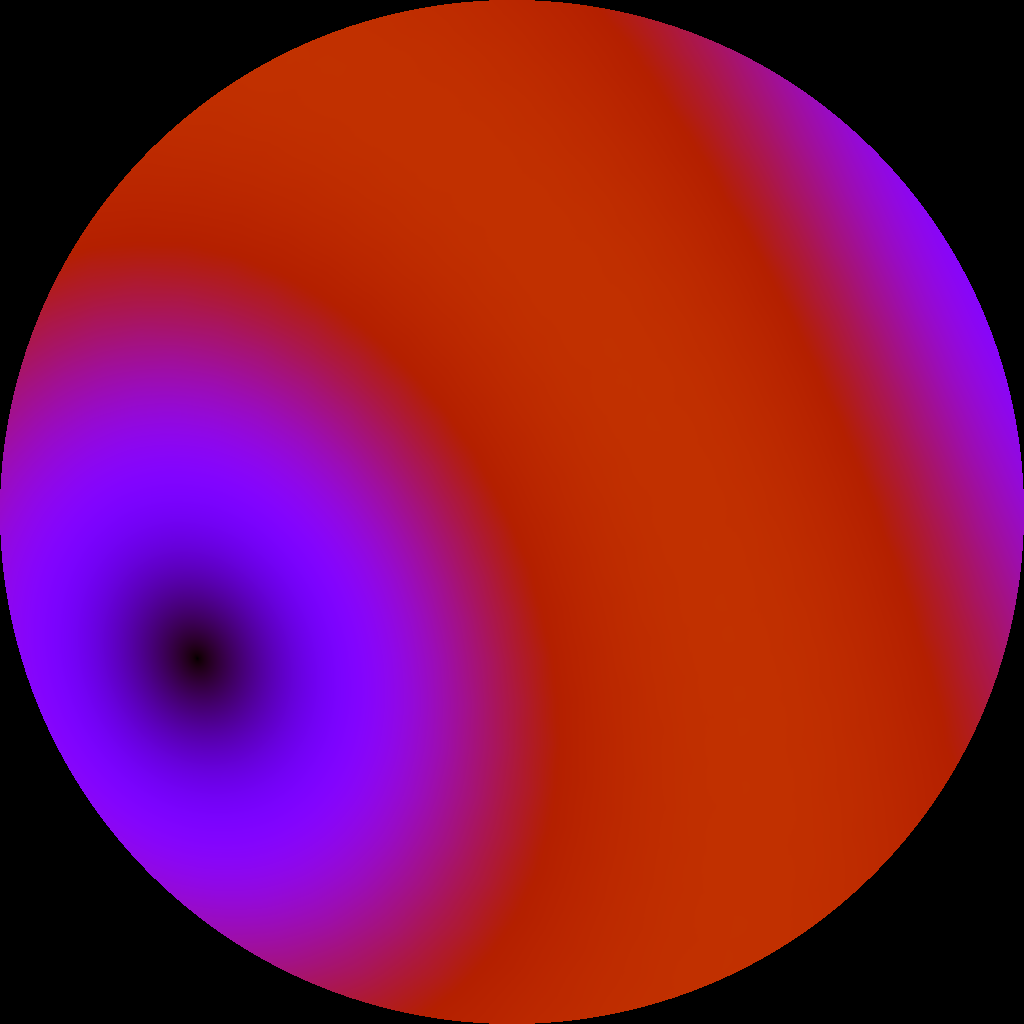

Supplement: Figure 6—source data 1. [file elife-70838-fig6-data1.zip › 6C/RightEye.png]

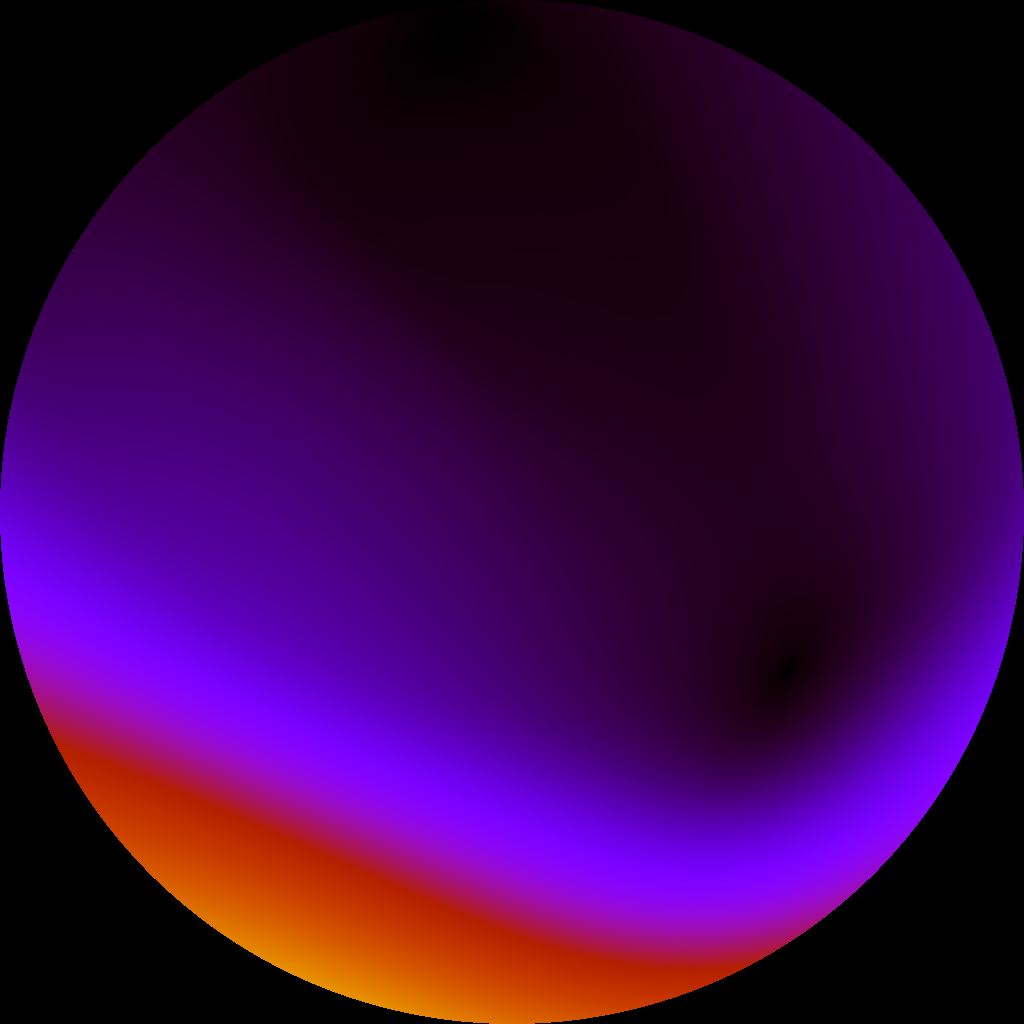

Supplement: Figure 6—source data 2. [file elife-70838-fig6-data2.zip › 6D/LeftEye.png]

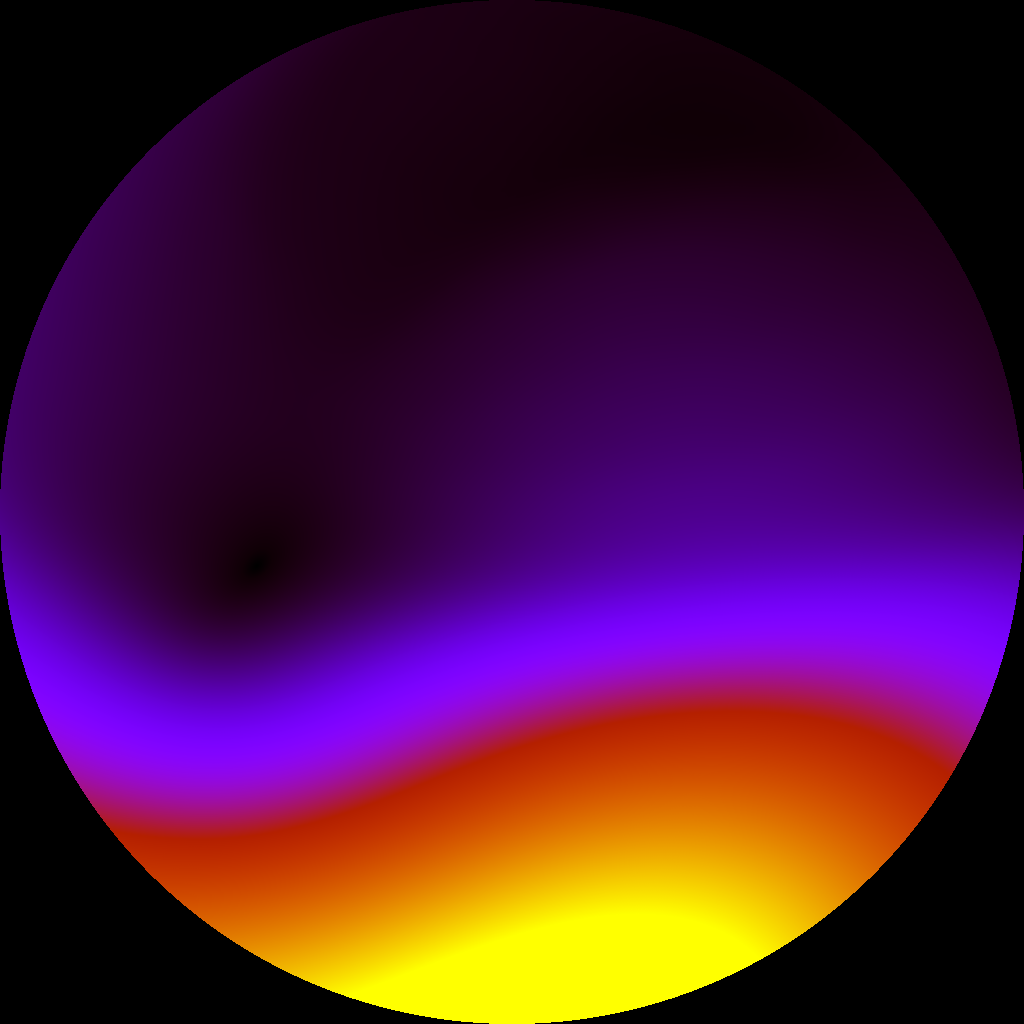

Supplement: Figure 6—source data 2. [file elife-70838-fig6-data2.zip › 6D/RightEye.png]

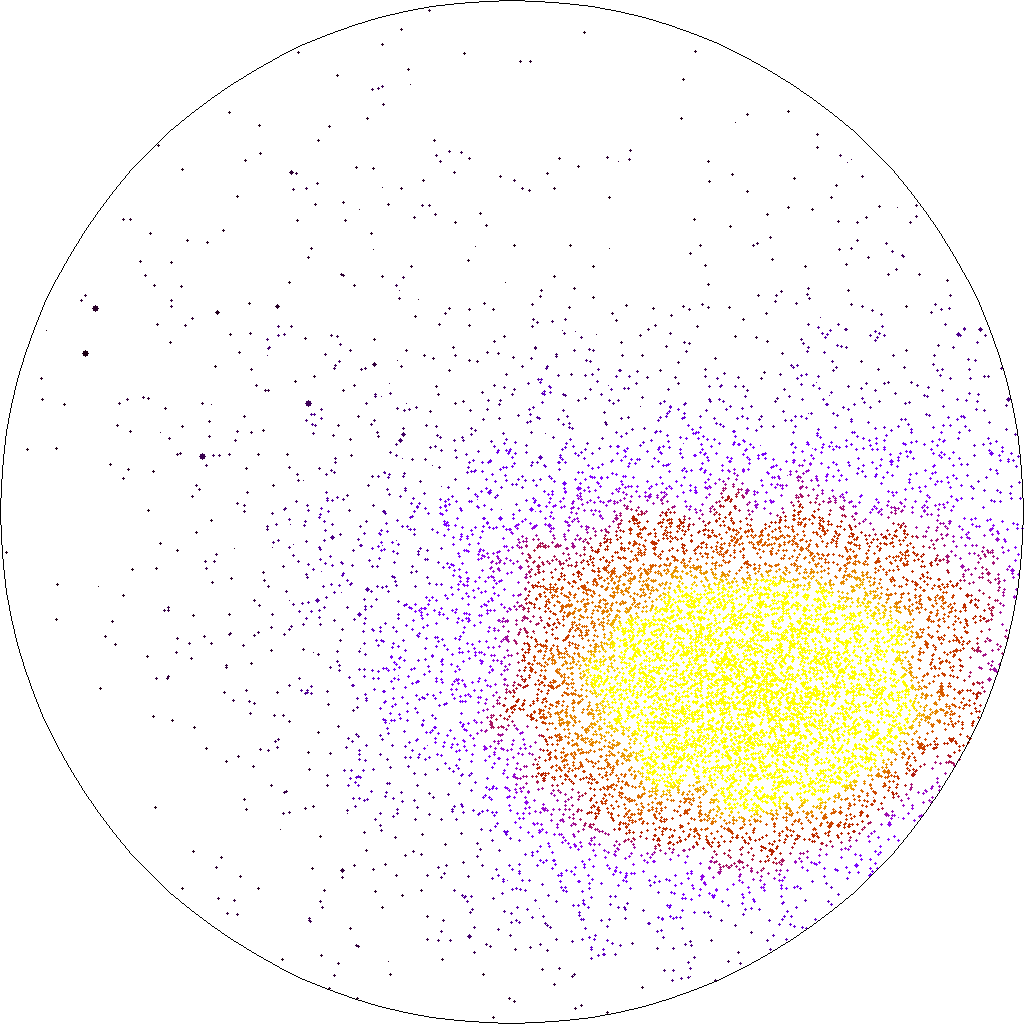

Supplement: Figure 6—source data 3. [file elife-70838-fig6-data3.zip › 6E/left.png]

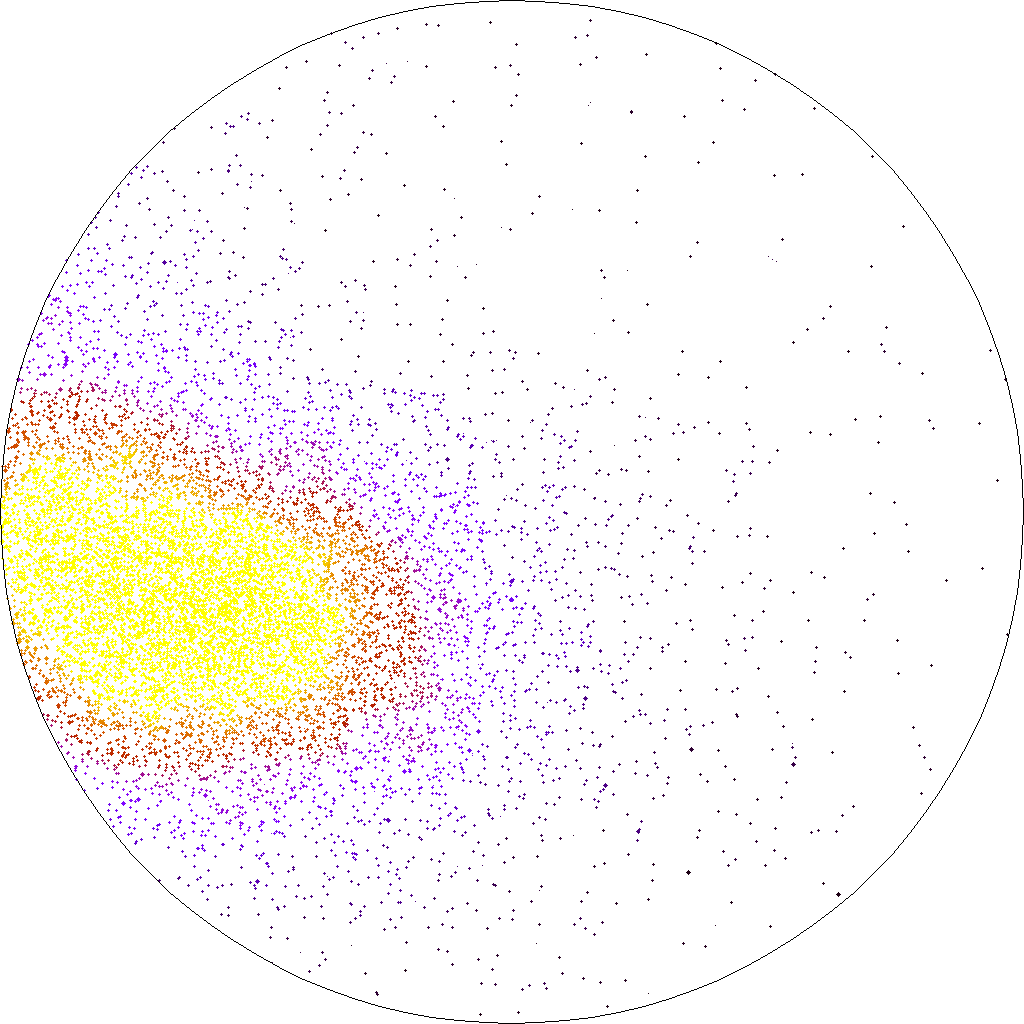

Supplement: Figure 6—source data 3. [file elife-70838-fig6-data3.zip › 6E/right.png]
