# Supplementary material for: Visual pursuit behavior in mice maintains the pursued prey on the retinal region with least optic flow
Source: eLife. 2021 Oct 26;10:e70838. doi: 10.7554/eLife.70838 (PMC8547958; doi:10.7554/eLife.70838)
Supplement: Transparent reporting form [file elife-70838-transrepform.docx]

***eLife’s* transparent reporting form**

We encourage authors to provide detailed information *within their submission* to facilitate the interpretation and replication of experiments. Authors can upload supporting documentation to indicate the use of appropriate reporting guidelines for health-related research (see [EQUATOR Network](http://www.equator-network.org/%20)), life science research (see the [BioSharing Information Resource](https://biosharing.org/)), or the [ARRIVE guidelines](http://www.plosbiology.org/article/info:doi/10.1371/journal.pbio.1000412) for reporting work involving animal research. Where applicable, authors should refer to any relevant reporting standards documents in this form.

If you have any questions, please consult our Journal Policies and/or contact us: [editorial@elifesciences.org](mailto:editorial@elifesciences.org).

**Sample-size estimation**

- You should state whether an appropriate sample size was computed when the study was being designed
- You should state the statistical method of sample size computation and any required assumptions
- If no explicit power analysis was used, you should describe how you decided what sample (replicate) size (number) to use

Please outline where this information can be found within the submission (e.g., sections or figure legends), or explain why this information doesn’t apply to your submission:

- Sample sizes for each of the values in text or figures can be found in the results section, figure and table legends and Methods.
- No explicit power analysis was used and sample sizes were not calculated in advance.
- Experiments were performed on a cohort of five animals, of which all required analyses could be performed on three.
- The experimental plan was to perform as many individual trials (prey captures) as the animals were motivated to perform.

**Replicates**

- You should report how often each experiment was performed
- You should include a definition of biological versus technical replication
- The data obtained should be provided and sufficient information should be provided to indicate the number of independent biological and/or technical replicates
- If you encountered any outliers, you should describe how these were handled
- Criteria for exclusion/inclusion of data should be clearly stated
- High-throughput sequence data should be uploaded before submission, with a private link for reviewers provided (these are available from both GEO and ArrayExpress)

Please outline where this information can be found within the submission (e.g., sections or figure legends), or explain why this information doesn’t apply to your submission:

- The total number of animals used in the study is provided in the text and methods section.
- Data relating to the number of animals used in each part of the study is given in the text, figure legends, and table legends.
- The number of trials is also given in the results section and in the figure legends. Where appropriate the number of frames used is given in the results section, and/or figure and table legends.
- Where trials (individual prey captures) have been broken down into segments the details of the number of segments has been provided in the results, figure legends and/or tables.
- Criteria for inclusion or exclusion of data and data sequences are given in the Methods section

**Statistical reporting**

- Statistical analysis methods should be described and justified
- Raw data should be presented in figures whenever informative to do so (typically when N per group is less than 10)
- For each experiment, you should identify the statistical tests used, exact values of N, definitions of center, methods of multiple test correction, and dispersion and precision measures (e.g., mean, median, SD, SEM, confidence intervals; and, for the major substantive results, a measure of effect size (e.g., Pearson's r, Cohen's d)
- Report exact p-values wherever possible alongside the summary statistics and 95% confidence intervals. These should be reported for all key questions and not only when the p-value is less than 0.05.

Please outline where this information can be found within the submission (e.g., sections or figure legends), or explain why this information doesn’t apply to your submission:

- Raw data is presented as individual examples in the main figures and/or in the figure supplements, and where possible figure panels and graphs contain all data points.
- Values of N (animals, trials and/or frames are reported in the text and figure legends).
- Both parametric and non-parametric statistics have been used in the study dependent on the characteristics of the distributions of the data in question.
- Whether the value given is the mean, median, SD, or Pearson’s r is given in the text and figure legends.
- Exact p-values are given for all statistical tests, and are stated in the results section and table and figure legends.
- If a figure panel includes an asterisk (or asterisks) as an indicator of a statistically significant difference between two groups of data, the exact p-values for the same figure panel can be found in the accompanying figure legend.

(For large datasets, or papers with a very large number of statistical tests, you may upload a single table file with tests, Ns, etc., with reference to sections in the manuscript.)

**Group allocation**

- Indicate how samples were allocated into experimental groups (in the case of clinical studies, please specify allocation to treatment method); if randomization was used, please also state if restricted randomization was applied
- Indicate if masking was used during group allocation, data collection and/or data analysis

Please outline where this information can be found within the submission (e.g., sections or figure legends), or explain why this information doesn’t apply to your submission:

N/A

**Additional data files (“source data”)**

- We encourage you to upload relevant additional data files, such as numerical data that are represented as a graph in a figure, or as a summary table
- Where provided, these should be in the most useful format, and they can be uploaded as “Source data” files linked to a main figure or table
- Include model definition files including the full list of parameters used
- Include code used for data analysis (e.g., R, MatLab)
- Avoid stating that data files are “available upon request”

Please indicate the figures or tables for which source data files have been provided:

- the mouse and cricket tracking data and resources for generating the reconstructed eye views will be available soon at a Dyrad repository.
- Source data files provided for Figure 1(D,G,H,I,J), Figure 2(A, B, C, D, E, F, G, H), Figure 3(A,B,C,D), Figure 4(B, C,D,G,H,I,J), Figure 5(A-G), Figure 6(C-E) and Figure 7(C&E).
